# Supplementary material for: Pyruvate Dehydrogenase Kinase 1 inhibition mediated oxidative phosphorylation enhancement in cartilage promotes osteoarthritis progression
Source: BMC Musculoskelet Disord. 2023 Jul 20;24:597. doi: 10.1186/s12891-023-06585-6 (PMC10357736; doi:10.1186/s12891-023-06585-6)
Supplement: Supplementary file 1 — Additional file 1. [file 12891_2023_6585_MOESM1_ESM.zip › BP.Gsea.1653623667859/GOBP_VACUOLAR_ACIDIFICATION.html]

Details for gene set GOBP\_VACUOLAR\_ACIDIFICATION[GSEA]

|  || Dataset | 55457\_collapsed\_to\_symbols.cls.cls #OA\_versus\_normal.cls.cls #OA\_versus\_normal\_repos |
| Phenotype | cls.cls#OA\_versus\_normal\_repos |
| Upregulated in class | OA |
| GeneSet | GOBP\_VACUOLAR\_ACIDIFICATION |
| Enrichment Score (ES) | 0.5466096 |
| Normalized Enrichment Score (NES) | 1.7375227 |
| Nominal p-value | 0.006960557 |
| FDR q-value | 0.12170375 |
| FWER p-Value | 0.998 |
Table: GSEA Results Summary

  

Fig 1: Enrichment plot: GOBP\_VACUOLAR\_ACIDIFICATION      
 Profile of the Running ES Score & Positions of GeneSet Members on the Rank Ordered List

  

| SYMBOL | TITLE | RANK IN GENE LIST | RANK METRIC SCORE | RUNNING ES | CORE ENRICHMENT || 1 | ATP6V0D1 | ATPase H+ transporting V0 subunit d1 [Source:HGNC Symbol;Acc:HGNC:13724] | 135 | 0.872 | 0.1403 | Yes |
| 2 | CLN5 | CLN5 intracellular trafficking protein [Source:HGNC Symbol;Acc:HGNC:2076] | 248 | 0.785 | 0.2674 | Yes |
| 3 | ATP6V0E2 | ATPase H+ transporting V0 subunit e2 [Source:HGNC Symbol;Acc:HGNC:21723] | 532 | 0.647 | 0.3572 | Yes |
| 4 | PPT1 | palmitoyl-protein thioesterase 1 [Source:HGNC Symbol;Acc:HGNC:9325] | 1042 | 0.508 | 0.4057 | Yes |
| 5 | ATP6V0E1 | ATPase H+ transporting V0 subunit e1 [Source:HGNC Symbol;Acc:HGNC:863] | 1207 | 0.474 | 0.4750 | Yes |
| 6 | ATP6V1H | ATPase H+ transporting V1 subunit H [Source:HGNC Symbol;Acc:HGNC:18303] | 1304 | 0.457 | 0.5466 | Yes |
| 7 | DMXL1 | Dmx like 1 [Source:HGNC Symbol;Acc:HGNC:2937] | 2766 | 0.273 | 0.4804 | No |
| 8 | GRN | granulin precursor [Source:HGNC Symbol;Acc:HGNC:4601] | 2962 | 0.254 | 0.5092 | No |
| 9 | TMEM106B | transmembrane protein 106B [Source:HGNC Symbol;Acc:HGNC:22407] | 3418 | 0.209 | 0.5101 | No |
| 10 | CLN6 | CLN6 transmembrane ER protein [Source:HGNC Symbol;Acc:HGNC:2077] | 3678 | 0.188 | 0.5225 | No |
| 11 | ATP6V0A1 | ATPase H+ transporting V0 subunit a1 [Source:HGNC Symbol;Acc:HGNC:865] | 4371 | 0.123 | 0.4900 | No |
| 12 | ATP6AP2 | ATPase H+ transporting accessory protein 2 [Source:HGNC Symbol;Acc:HGNC:18305] | 5203 | 0.056 | 0.4352 | No |
| 13 | ATP6V0A4 | ATPase H+ transporting V0 subunit a4 [Source:HGNC Symbol;Acc:HGNC:866] | 5968 | -0.002 | 0.3763 | No |
| 14 | DMXL2 | Dmx like 2 [Source:HGNC Symbol;Acc:HGNC:2938] | 6655 | -0.053 | 0.3323 | No |
| 15 | SLC11A1 | solute carrier family 11 member 1 [Source:HGNC Symbol;Acc:HGNC:10907] | 7153 | -0.092 | 0.3096 | No |
| 16 | TCIRG1 | "T cell immune regulator 1, ATPase H+ transporting V0 subunit a3 [Source:HGNC Symbol;Acc:HGNC:11647]" | 7600 | -0.127 | 0.2970 | No |
| 17 | CLN3 | "CLN3 lysosomal/endosomal transmembrane protein, battenin [Source:HGNC Symbol;Acc:HGNC:2074]" | 8094 | -0.163 | 0.2869 | No |
| 18 | ATP6V0A2 | ATPase H+ transporting V0 subunit a2 [Source:HGNC Symbol;Acc:HGNC:18481] | 8843 | -0.221 | 0.2670 | No |
| 19 | CLIC4 | chloride intracellular channel 4 [Source:HGNC Symbol;Acc:HGNC:13518] | 9537 | -0.277 | 0.2611 | No |
Table: GSEA details [plain text format]

  

Fig 2: GOBP\_VACUOLAR\_ACIDIFICATION      
 Blue-Pink O' Gram in the Space of the Analyzed GeneSet

  

Fig 3: GOBP\_VACUOLAR\_ACIDIFICATION: Random ES distribution      
 Gene set null distribution of ES for **GOBP\_VACUOLAR\_ACIDIFICATION**
